# Dynamic and social behaviors of human pluripotent stem cells

**DOI:** 10.1038/srep14209

**Published:** 2015-09-18

**Authors:** Smruti M. Phadnis, Nathan O. Loewke, Ivan K. Dimov, Sunil Pai, Christine E. Amwake, Olav Solgaard, Thomas M. Baer, Bertha Chen, Renee A. Reijo Pera

**Affiliations:** 1 Institute for Stem Cell Biology and Regenerative Medicine, School of Medicine, Stanford University, California, USA; 2 Department of Genetics, School of Medicine, Stanford University, California, USA; 3 Department of Obstetrics and Gynecology, School of Medicine, Stanford University, California, USA; 4 Department of Electrical Engineering, Stanford University, Stanford, California, USA; 5 Edward L. Ginzton Laboratory, Stanford University, Stanford, California, USA; 6 Stanford Photonics Research Center, Department of Applied Physics, Stanford University, Stanford, California, USA

## Abstract

Human pluripotent stem cells (hPSCs) can self-renew or differentiate to diverse cell types, thus providing a platform for basic and clinical applications. However, pluripotent stem cell populations are heterogeneous and functional properties at the single cell level are poorly documented leading to inefficiencies in differentiation and concerns regarding reproducibility and safety. Here, we use non-invasive time-lapse imaging to continuously examine hPSC maintenance and differentiation and to predict cell viability and fate. We document dynamic behaviors and social interactions that prospectively distinguish hPSC survival, self-renewal, and differentiation. Results highlight the molecular role of E-cadherin not only for cell-cell contact but also for clonal propagation of hPSCs. Results indicate that use of continuous time-lapse imaging can distinguish cellular heterogeneity with respect to pluripotency as well as a subset of karyotypic abnormalities whose dynamic properties were monitored.

Previous studies demonstrated that non-invasive imaging of cell cycle parameters provides a useful tool to prospectively predict developmental success or failure that is linked to genetic stability in preimplantation human embryos^1^,^2^. Human pluripotent stem cells (hPSCs) can be derived either from human embryos or alternatively by reprogramming somatic cells to an embryonic stem cell-like fate^3^,^4^. Although recent advances in single cell analyses have demonstrated remarkable heterogeneity in hPSC populations^5^, our understanding of individual pluripotent stem cells remains limited. Limitations are largely due to technical hurdles that include invasive retrospective tests for stem cell function, low differentiation efficiencies and asynchrony in cell cycle progression. Long term live cell imaging and quantitative analyses of the dynamics of cell populations may help overcome current limitations and complement invasive analytical techniques^6^.

In this study, we developed non-invasive methods to reliably predict fate of hPSCs and their differentiated progeny via time-lapse microscopy. We hypothesized that distinct stem cell behaviors are diagnostic of self-renewing cells, differentiated progeny and potentially, although not yet explored, disease, genetic and/or epigenetic status. We show here that hPSCs in culture display unique dynamic behavioral patterns that can be measured and quantified. We anticipate that observation of social and dynamic behavior of hPSCs may provide an additional means for routine assessment of stem cells for basic and pre-clinical applications to insure reproducibility, safety and/or efficacy.

## Results

### Pluripotent cells exhibit dynamic behavior

**Figure 1:**
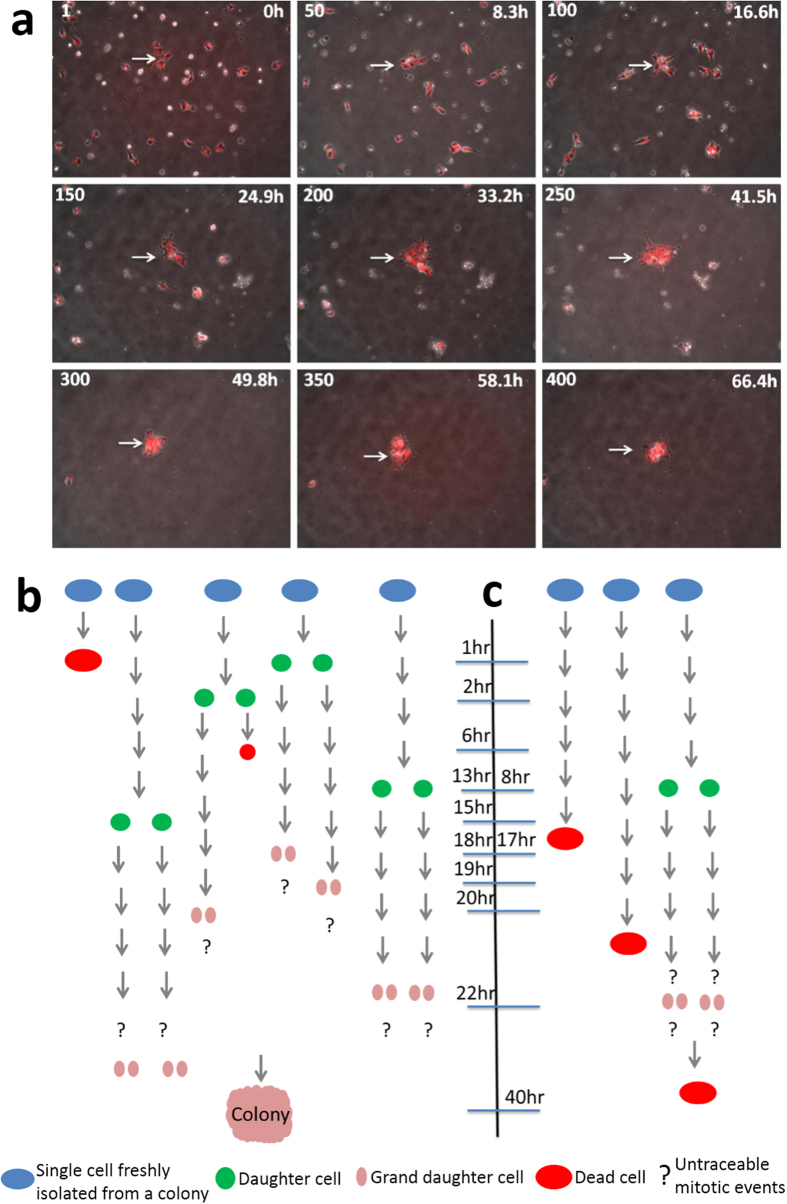
Continuous monitoring of human embryonic stem cells via time-lapse imaging. (**a**) Image of a field of human embryonic stem cells (hESCs) seeded at 1,500 cells/cm^2^ over 3 days. (**b**) Manual analysis of the cells forming colonies and (**c**) cells failing to form colonies. We observed that production of granddaughter cells within 24 hrs of plating was strongly correlated with successful colony formation.

To evaluate whether quantitative, non-invasive methods of analyzing cell behavior during self-renewal and differentiation of human embryonic stem cells (hESCs) might allow prediction of cell state and outcomes, we began by focusing on the dynamics of colony formation. Single cells derived from hESC colonies were first labeled with CDy1, a fluorescent rosamine dye which specifically labels pluripotent cells^7^,^8^, and then were plated on matrigel coated plates at different densities (150,000; 15,000 and 1,500 cells/cm^2^). Cell image data was acquired continuously for over 96 h (Supplementary Fig. S1a and Supplementary movies 1, 2, 3). As shown in Supplementary Fig. S1c, poor survival of the cells was observed at low densities, as previously reported^9^. We then used customized semi-automated tracking software termed the Cell Moment Tracker (CMT, Supplementary Fig. 2 and supplementary movie 4a, 4b) to extract distinct changes in cell cycle lengths that depended upon seeding density. Cells seeded at higher density (were tracked manually), had shorter cell cycle times and higher mitotic rates relative to those seeded at mid- and low-density (Supplementary Fig. S1b). We also observed that cells seeded at low densities extended more cellular appendages towards neighboring cells, thus increasing both their cross-sectional (cellular) area and volume. In contrast, cells at high density were more compact and aggregated efficiently with neighbors, thus contributing to colony formation. Notably, cells at low densities (1,500cells/cm^2^) showed greater variability in cell behavior. Nonetheless, cell behaviors could be quantified and single cells were tracked. For the remaining experiments, we seeded cells at low density (Fig. 1a, Supplementary movies 3a & 4a).

We scored cells based on their ability to form colonies. By manually counting and tracking cells, we observed that it is critical for survival that a small number of cells initiate colony formation. As shown in Fig. 1b, when three or more cells associate closely and give rise to granddaughter cells, within the first 24 hrs post-plating, a pluripotent colony is formed. If the cells fail to divide (Fig. 1c) or leave the group within this time period, then colony formation is unlikely. Based on these results, we analyzed the data generated via CMT and observed that the average distance between cells that form colonies is <50 um. Cells that migrate and ultimately supplement colony formation are within 50–200 um and cells that fail to contribute to colonies are beyond 300 um. We termed cells that contribute to colony formation as ‘neighbor’ cells when within 100 um of cells of interest. We also observed that the migration of individual cells differed between cells that contributed to colonies and those that did not. These observations corroborate the ‘community effect’ as described theoretically by Saka *et al.*^10^. as an interaction among a group of nearby cells which is necessary to form or maintain a tissue.

**Figure 2:**
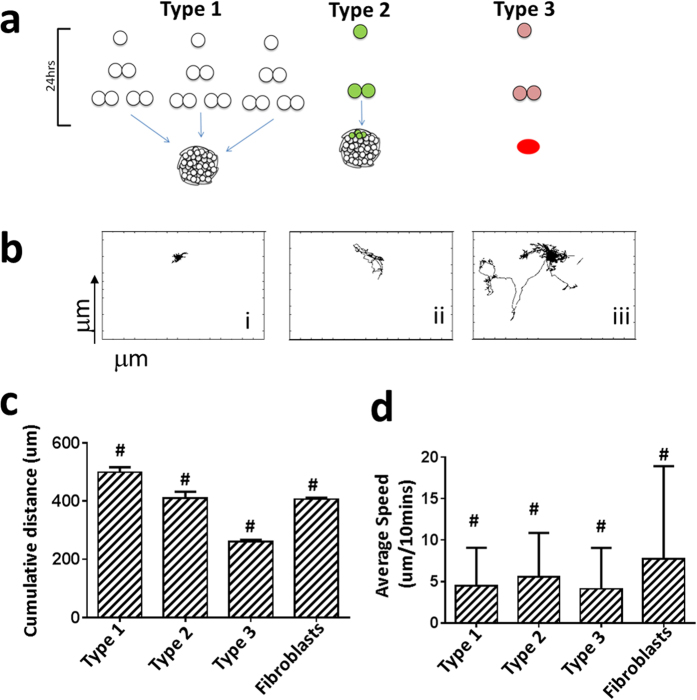
Characterization of cells based on dynamic properties. (**a**) Classification of singe cells derived from hESC colonies based on their dynamic properties. Cells that form colonies (Type 1), cells that support colony formation (Type 2), and cells which were unable to form colonies (Type 3) (n = 48 fields of view, with 3 different sets of data). (**b**) Representative migratory behavior of Type 1, 2, and 3 cells when cultured at 1,500 cells/cm^2^. (**c**) Cumulative distance travelled by Type 1, 2, and 3 cells (n ~ 100–150) compared with human BJ fibroblasts (n = 100). (**d**) Average speed of cells (n ~ 100–150 cells each) tracked through survival and/or merge into a colony (24–48 hrs). ^#^indicates Two Way Anova; p < 0.0001.

Based on observations described above and data generated through CMT (Supplementary Movies 3b & 4b), we concentrated analysis on the dynamic properties that cells exhibit during colony formation including speed of movement, mitotic events, total distance travelled, area and distance to nearest neighbors. We observed three types of distinct cell behaviors that define hPSCs (Supplementary Fig. S3). Cells that form a colony by themselves (either by clonal propagation or by aggregating with nearby cells) are termed Type 1 (colony forming cells) (Fig. 2a and Supplementary movie 4b). Cells that extend appendages and demonstrate migratory behavior to ultimately fuse or join colonies are termed Type 2 (colony fusing cells) and single cells that do not form or supplement colonies are termed Type 3 (non-colony forming cells). Type 1 cells demonstrated directed migration patterns and less displacement in terms of speed relative to Type 2 cells which displayed random and selection-based movements or Type 3 cells which demonstrated random migratory patterns and traveled a large distance (Fig. 2b,d). Notably, Type 1 cells were characterized by greater cumulative migratory distance even though they traveled a shorter distance than Type 2 and 3 cells (Fig. 2c). This indicated that the cells involved in early colony formation are not stationary and may play a critical role in early colony organization.

### Pluripotent cell interactions with neighbors

**Figure 3:**
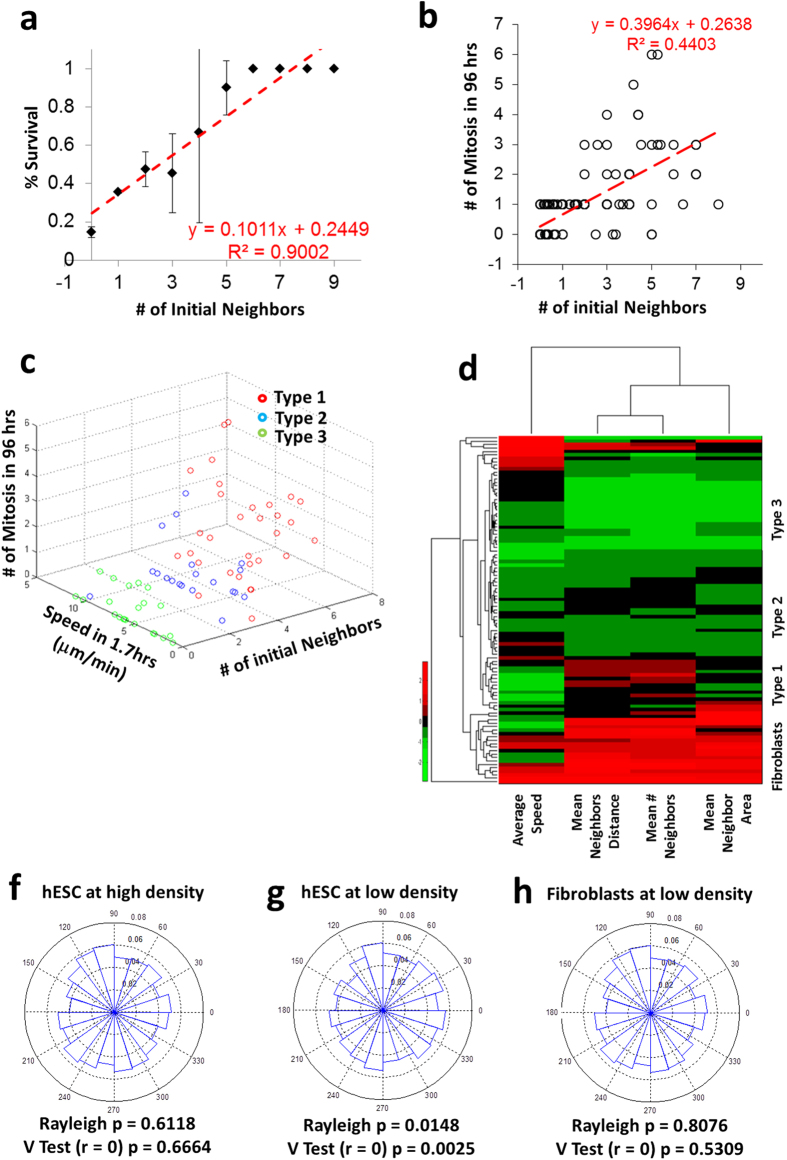
Influence of neighbors on isolated hESCs. (**a**) Correlation of survival of cells and (**b**) number of mitotic events with initial number of neighbors (n ~ 20 to 40 cells). (**c**) 3D plot depicting the segregation of the three types of cells based on initial number of neighbors, speed and number of mitotic events (n ~ 20 to 40 cells). (**d**) Heat map denoting clustering of cells based on neighbor properties for hESCs (n ~ 100) and human BJ fibroblasts (n = 20). Radial histogram characterizing cell migration based on proximity to neighbors (probability of the frequency of vectors in each bin) and verified with Rayleigh test and V test for hESCs seeded at (**f**) 15,000 cells/cm^2^ and (**g**) 1,500 cells/cm^2^, and for (**h**) human BJ fibroblasts seeded at 1,500 cells/cm^2^ (n ~ 100).

Given that survival of single cells is compromised at low density (Supplementary Fig. 1c), we hypothesized that interactions between neighboring cells might be a key characteristic of pluripotent stem cells. We analyzed the initial number of neighbors (number of neighbors at plating or time 0) in the three population types and observed that Type 1 cells had more neighbors than Type 2 or Type 3 cells (Supplementary Fig. S3a). We then tested whether there was an effect of neighbors on cell survival and mitosis and found that survival is highly correlated with the initial number of neighbors, with survival rate increasing as density of the cells increased (Fig. 3a). Mitotic events also correlated with initial number of neighbors (Fig. 3b), indicating the importance of neighboring cells in the colony forming process. When average speed, number of mitotic events and number of initial neighbors were plotted, a clear segregation of the three types of cells was observed (Fig. 3c).

To further investigate neighbor behavior and determine how neighbor size and mean distance from the cells could impact survival, we generated clustered heat maps of neighbor properties for cells that were tracked. We observed that Type 1, 2 and 3 cells clustered based solely on neighbor features (Fig. 3d). Interestingly we also observed that Type 3 cells show larger displacement than Type 1 cells (Fig. 2bi, iii), though their cumulative distance travelled is less than Type1 cells (Fig. 2c). Hence their distance to neighbors is variable in time and when seen collectively over time, show shorter neighbor distances than Type1 cells. Type 2 cells demonstrated an intermediate phenotype in respect to neighbor properties. As a control, somatic cells (fibroblasts) were analyzed similarly and showed no evidence of neighbor dependence or similar dynamic behaviors (Fig. 3d, Supplementary movie 5).

Neighbor interactions suggest that cells may sense their neighbors via cytokine or chemokine attractions with migration typified by chemotaxis^11^. In order to test this concept, we calculated the direction of each cell’s nearest neighbor for every frame in the time-lapse sequence. We hypothesized that if every cell secretes cytokine or chemokine signals, then from the perspective of a lone single cell, the gradient would be strongest towards the nearest neighbor, thus biasing migration toward that neighbor. To determine if this is the case, we analyzed the distribution of the direction of the instantaneous displacement vector of every cell at every frame. The direction of displacement vectors was calculated with respect to the direction of the nearest neighbor. Thus, displacement in the direction of 0 [degrees] indicates movement towards the nearest neighbor. In order to determine if there was any bias in the direction of data points, we employed the Rayleigh test^11^. The rejection of the null hypothesis by the Rayleigh test indicates unimodal clustering of cell directions. In addition, we used the V test which allows the *a priori* specification of an expected direction^12^. Rejection of the null hypothesis in the V test would suggest that there is bias in a specific direction. As seen in the radial histogram plots, hESCs seeded at high density (Fig. 3f), displayed little or no chemotactic migration in any one direction. In contrast, cells that were seeded at lower density displayed directional migration toward the nearest neighbors (at 0 degrees in the plot) (Fig. 3g). Notably, control fibroblasts showed no directional migration (Fig. 3h), thus highlighting differences in pluripotent cells and differentiated cells.

Based on the above findings, we summarize likely early events of colony formation from single cells dissociated from a parent colony as follows: 1) Extracellular matrix (ECM) recognition and attachment. 2) Sensing of neighbors and surroundings via: i. attraction - cytokines, chemokines, adhesion molecules, ECM-degrading proteases, haptotaxis (contact guidance) and ii. migration, typified by more directional and organized when cells fuse into colonies, biased when surrounded by neighbors, random with no sense of direction when isolated. 3) Aggregation of neighboring cells. 4) Expansion of colonies with an increase in cell cycle time indicative of growth of cell populations.

### E-cadherin/TRA160 dual positive single cells show clonal propagation

**Figure 4:**
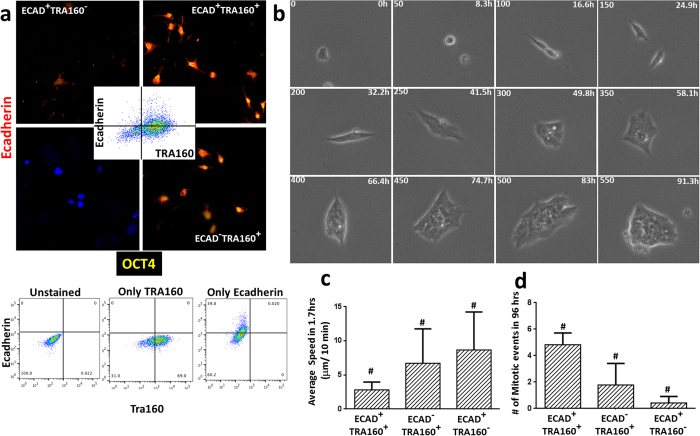
E-cadherin expression drives the dynamic behavior of hESCs. (**a**) Representative FACS profile of hESCs based on E-cadherin and TRA160 (inset) expression revealed that not all TRA160 positive cells express membrane bound E-cadherin. FACs profile for controls used for gating. (**b**) Snapshot of E-cadherin and TRA160 positive cells showing clonal propagation. The three phenotypes showed similar dynamic behavior with respect to average speed (**c**) and number of mitotic events (**d**) to the cells classified based on imaging properties as shown in Fig. 1. ^#^indicates Two Way Anova; P < 0.05 and 0.0001, respectively.

E-cadherin (ECAD), a transmembrane protein that plays an important role in cell adhesion, is known to have an important function in maintaining pluripotency of hPSCs and in reprogramming of fibroblasts into iPSCs^13^,^14^. To explore whether ECAD might be implicated in propagation of hPSCs, we immunostained single hESCs for ECAD protein expression and observed that although most cells expressed membrane-bound ECAD, some cells exhibited intracellular ECAD localization (data not shown). Hence, we labeled single hESCs with TRA160 (a pluripotent marker) and ECAD and analyzed the sorted population. We observed that although ~80% of cells expressed TRA160, only half of that population had membrane bound ECAD protein expression (Fig. 4a (inset)). We sorted the cells based on their ECAD and TRA160 expression and seeded them at a density of 1,500 cells/cm^2^, and followed them via time-lapse microscopy. Results indicated that the majority of cells that were ECAD^+^TRA160^+^, expressed membrane bound Ecadherin and nuclear OCT4; demonstrated ability to clonally propagate and form colonies within 4–5 days (Fig. 4a,b and Supplementary movie 6). In contrast, ECAD^**−**^TRA160^**+**^cells demonstrated nuclear OCT4 along with Ecadherin localized either in the cytoplasm or in the nucleus (Fig. 4a) and were dependent on neighbors to form pluripotent colonies, whereas ECAD^**+**^TRA160^**−**^ cells failed to form any colonies. Dual positive cells also demonstrated greater numbers of mitotic events than the other two types of cells (Fig. 4d). When we compared the dynamic properties of the ECAD^**+**^TRA160^**+**^, ECAD^**−**^TRA160^**+**^ and ECAD^**+**^TRA160^**−**^ cells with an emphasis on migration speed, we observed that these three phenotypes reflected migratory behavior of Type 1, 2 and 3 cells, respectively (Supplementary Fig. S3b). These findings indicate that phenotypic and dynamic analysis of cell behavior relays information regarding molecular status with potential to predict fate of the cells.

### hPSCs can retain pluripotency and interact when cultured in somatic cell culture media

**Figure 5:**
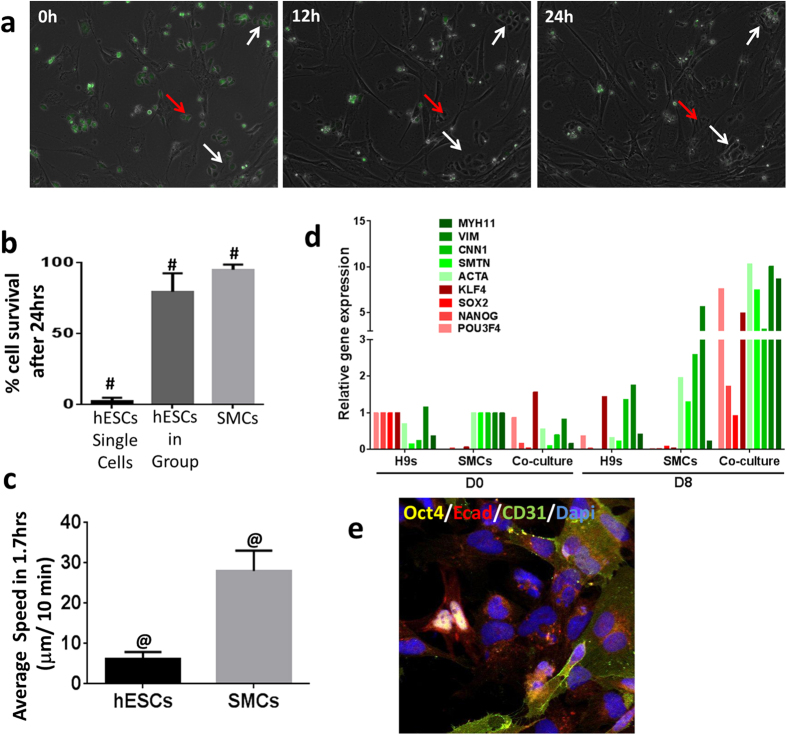
Socially-networked hPSCs still retain their pluripotency in somatic cell medium. (**a**) Co-culture of hESCs and smooth muscle cells (SMCs) in SMC growth medium. hESCs were labeled with CFSE dye (green) and mixed with SMCs at a 5:1 ratio. White arrows indicate hESCs in clumps and red arrows indicate single hESCs. (**b**) Percentage of cells that survived after 24 hrs of seeding. (**c**) Average speed of cells in first 100 mins after attachment. (**d**) Relative gene expression of pluripotent markers (POU3f4, NANOG, SOX2, KLF4) and SMC markers (ACTA, SMTN, CNN1, VIM, MYH11) with respect to Day 0 expression of hESCs and SMCs respectively. (**e**) Representative image of Day 8 co-culture of hESCs and SMCs showing active expression of Oct4 (yellow) and E-cadherin (red). ^#^indicates Two Way Anova; P < 0.0005 and 0.0001, respectively.

Basic fibroblast growth factor (bFGF) can support feeder-independent growth of hESCs at high concentrations^15^. Removal or reduced concentrations of bFGF results in loss of pluripotency. For example, in our studies, we used mTeSR1 media to culture hPSCs. This media contains 100 ng/ml of bFGF, whereas, somatic cell growth media such as SMGS (Smooth Muscle cell growth supplement) contains only 2 ng/ml bFGF. To examine behaviors of hPSCs in culture with different bFGF concentrations, we co-cultured single cells and small groups of 5–7 hESCs with smooth muscle cells (SMCs) for a period of 8 days in SMGS. For ease of identification, we labeled the hESCs with CFSE dye. As shown, we tracked single and small clumps of hESCs and observed that single isolated hESCs showed very little cell survival in SMGS relative to hESCs in groups (Fig. 5a,b and Supplementary Movie 7). We also observed that SMCs demonstrated greater migrational speeds than hESCs in SMGS (Fig. 5c). Relative gene expression data (Fig. 5d) of Day 0 and Day 8 co-cultured cells indicated that both the hESC population and SMC population expanded over the course of 8 days. Furthermore, in the co-cultured group, we observed that SMCs act like feeder cells to the small clumps of hESCs thereby increasing the expression of pluripotency markers. However the hESCs alone in SMGS medium show decrease in the expression of pluripotency markers due to spontaneous differentiation in low bFGF conditions (Fig. 5d).

Indeed, hPSCs at Day 8 demonstrated strong expression of nuclear OCT4 and ECAD in cells that were tightly aggregated in small colonies (Fig. 5e). Expression of CD31 in the vicinity of the hESC colonies suggested that few cells spontaneously differentiated toward SMCs. These results demonstrated that hESCs with sufficient neighbors retained hallmarks of pluripotency and continued to proliferate even in somatic cell culture conditions. In contrast, groups of hESCs of less than 5–7 cells were not observed and most likely differentiated or died. Thus, the presence of neighbors is an important dynamic parameter that modulates cell fate since hPSCs with sufficient neighbors retain pluripotency and continued to proliferate even in the presence of media optimized for culture of somatic cells.

### Cell dynamics during early differentiation

We next examined cellular dynamics during early differentiation events by transferring cells to differentiation media and tracking morphological and behavioral changes for 72 hours. We compared early differentiation to spontaneous differentiation of hESCs to fibroblasts, a common fate observed in routine culturing of hESCs (Supplementary Fig. S5ai and Supplementary Movie 8) and following exposure to 20% fetal bovine serum (Supplementary Fig. S5aii and Supplementary movie 9). We observed that early differentiating cells behaved differently than hESCs. Rather than aggregating to form a tight colony, the cells accumulated cytoplasm, thereby increasing cell surface area (Supplementary Fig. S5b) and displayed altered mitotic parameters and increased cell cycle times (Supplementary Fig. S5c).

**Figure 6 f6:**
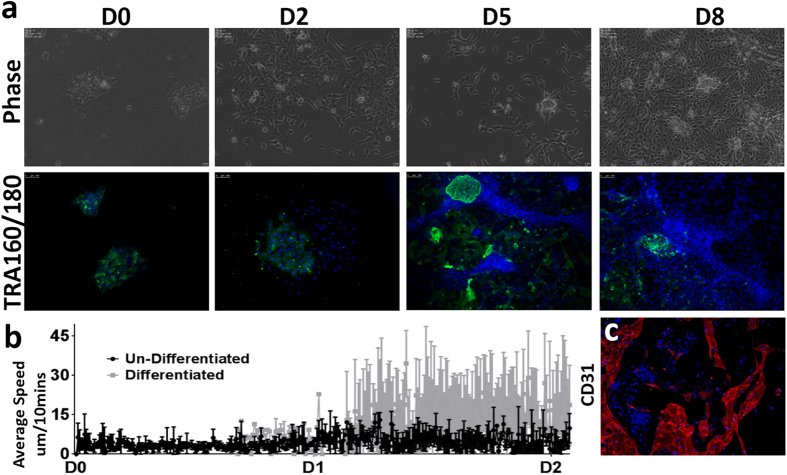
Dynamic behaviors during early differentiation of hESCs to vascular progenitors. (**a**) Time-lapse images of H9 hESCs undergoing differentiation to vascular progenitors (CD31^**+**^/34^**+**^ cells). Representative images of cells stained with TRA160/180 during differentiation. (**b**) Dynamic differences (in form of average speed) between differentiated cells and undifferentiated cells during early differentiation (n = 50–100 cells per group for 3 different fields of view). (**c**) CD31 expressing cells on Day 8 of differentiation.

In order to investigate the dynamic properties of hPSCs undergoing targeted differentiation, we exposed small colonies of 50–100 hESCs to chemically defined medium supplemented with factors that promote generation of vascular progenitors^16^. As shown in Fig. 6a (top panel, Supplementary Movie 10), we observed phenotypic changes similar to those observed with spontaneous differentiation: namely, an increase in cell surface area relative to hPSCs and a rapid decrease in frequency of mitotic events from D0 to D2. TRA160/180 staining over the course of differentiation of the cells revealed that many cells do not reduce expression of these markers of pluripotency immediately upon differentiation. Indeed, a subset of cells continued to proliferate and remained pluripotent even after eight days of differentiation. When we assessed the dynamic properties of differentiated as well as undifferentiated cells for the first two days of differentiation, we observed that differentiated cells showed higher migration speeds as compared to the undifferentiated cells (Fig. 6b). However, not all cells gave rise to vascular progenitors (CD31^+^ cells) and some cells continued to express TRA160/180 (Fig. 6a[Fig f6]c (lower panel)).

### Dynamic behaviors may reflect karyotype

**Figure 7:**
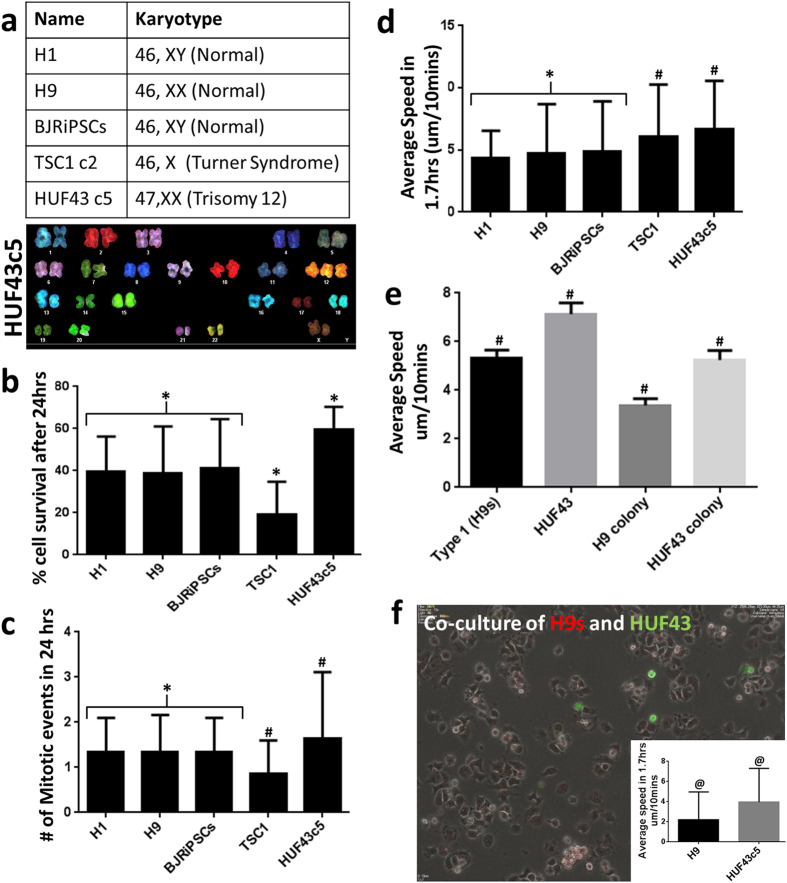
Dynamic behaviors of normal and abnormal karyotypic cells. (**a**) Cell lines and karyotypes analyzed. (**b**) Percentage of cells that survived after 24 hrs of seeding. (**c**) Number of mitotic events in 24 hrs following cell attachment. (**d**) Dynamic differences across all the karyotype in form of average speed within 100 mins of cell attachment when cells were seeded at density of 1,500 cells/cm^2^. (**e**) Average speed of single cells as well as colonies derived from H9 hESCs and HUF43c5 iPSCs over 3 days. (**f**) Co-culture of H9 hESCs and HUF43c5 iPSCs in the ratio of 5:1. HUF43c5 cells were labeled with CFSE and their dynamic differences (inset) in terms of speed. *Indicates no statistical difference, ^#^indicates Two Way Anova (P < 0.05) and @ indicates Student’s t test (p < 0.0001).

hPSCs are prone to genetic instablity^17^. For example, trisomy of chromosome 12 is often seen with continuous culture of pluripotent stem cells and in certain cancer cells^18^. Recent studies have shown that “adapted” cells that carry karyotypic abnormalities can display characteristic cell behaviors that differ from those of karyotypically normal hESCs^19^. These adapted cells can proliferate without neighbor interactions and independent of migratory patterns. We hypothesized that karyotypic abnormality might be detected at the single cell level and quantified through altered dynamic properties relative to cells with normal karyotypes and quantified via use of the CMT. Thus, we compared karyotypically-normal hESCs (H1 and H9) and iPSCs (BJRiPCs^20^) to those with two abnormal karyotypes (iPSCs (TSC1c2 – Turner Syndrome^21^)) and HUF43c5 (Trisomy 12) (Fig. 7a). When we used a similar experimental protocol as above and analyzed the data via the CMT, we observed that cell survival and number of mitotic events were similar across normal karyotypic cells. In contrast, TSC1c2 cells that lack a second X chromosome showed reduced cell survival after 24 hrs of plating and HUF43c5 cells, with an extra chromosome 12, showed higher cell survival (Fig. 7b). The number of mitotic events was also higher in HUF43c5 cells relative to TSC1c2 cells (Fig. 7c). Surprisingly, we observed that the single cells from abnormal karyotypic cells migrated over a larger distance, with enhanced motility (Fig. 7d). Further, when we compared the velocities to single hESCs, as well as those in small colonies, we observed a significant difference between the hESCs and the HUF43c5-iPSCs (Fig. 7e and Supplementary Movie 11). In order to detect karyotypic abnormalities, we co-cultured hESCs and HUF43c5 iPSCs in a 5:1 ratio and tracked the cells using the CMT. For ease of tracking, we labeled HUF43c5 cells with CFSE dye. We documented similar differences in the speed of movement in the two types of cells in co-cultures (Fig. 7f and Supplementary Movie 12). We stress that although this observation cannot be generalized to all abnormal karyotypic cells, it indicates that karyotypic abnormalities can alter dynamic characteristics and may be detectable and quantified via non-invasive methods.

## Discussion

Human development begins with a single cell embryo that gives rise to a variety of cell types in order to ultimately form a fully developed healthy individual. The behavior of cells in developing tissues and their interactions with other cell types define differentiation during development. Thus, documenting and quantifying behavior of individual cells may inform studies of fundamental human biology and provide methods to prospectively predict cellular responses of pluripotent stem cells and their differentiated progeny.

Our results suggest that hPSCs, both hESCs and iPSCs display dynamic behavioral patterns that can be measured through time-lapse imaging and use of automated, quantitative image analysis in order to predict the outcome of their next division via algorithmic statistics. Results indicate that migratory movement (directional to the neighbors) is a characteristic dynamic social behavior of hPSCs that is predictive of cell survival and maintenance of pluripotency. These results correlate well with previous studies that have shown that individual cell movement, asymmetric colony expansion, Rho-associated kinase, and ECAD may form a functional network that impacts hESC clonogenicity^9^. Here, we demonstrate that heterogeneity within a pluripotent colony can be identified using continuous time-lapse microscopy and that behaviors are well-correlated with presence or absence of biochemical markers. These findings coupled with gene expression data and protein profiles can be used to monitor cellular heterogeneity in real time (Supplementary Fig. S4).

An application of the research described here may be in use in prospectively validating and isolating cells of a pre-determined developmental potential. Results are transferrable to both hESCs and iPSCs (for normal karyotype as indicated in our results) and examination of subtle behaviors that may ultimately influence cellular outcomes *in vitro* or *in vivo.* A major roadblock to the use of pluripotent stem cells is the limitation of diagnostics and predictive capabilities at this time, with most assays being invasive (RNAseq, marker analysis and genetic/epigenetic analyses) and population-based. Practical and effective cell-quality assurance methods might benefit from analysis of cell behaviors and be used in conjunction with invasive methods to minimize variability in growth and differentiation protocols prior to basic and clinical applications. Thus, time lapse and quantitative imaging of human pluripotent stem cells during growth and differentiation may contribute to our repertoire of tools to understand and predict cell fate.

## Methods

### Cell Culture

Undifferentiated pluripotent stem cell lines, hESC lines: H9 (46, XX) and H1 (46, XY) and human derived iPSC lines: BJRiPCs (46, XY), HUF43 (46+1, XX), TSC1c2 (46-1, X) were maintained in feeder free conditions with mTeSR1 (Stem Cell Technologies, Vancouver, BC, Canada). For production of single cell suspension, colonies were pretreated with Rock inhibitor (Y-27632, Cellagen Technology) for 30 mins and then exposed to Accutase ((Innovative Cell Technologies, Inc, San Diego, CA) supplemented with Rock inhibitor for 2–3 mins. Accutase was then diluted ten-fold with Calcium – Magnesium free PBS and the suspension was gently flushed several times by 1 ml pipette tip. The suspension was centrifuged at 1000 rpm for 5 mins and the cells were counted and seeded in mTeSR supplemented with Thiazovivin (Santa Cruz Biotechnology). Cells were incubated for 30 mins at 37 ^°^C in 5% CO_2._ After changing the medium to plain mTeSR, the cells were taken for imaging. Medium was changed every 24 hours.

For labeling with CDy1 dye, we used methods as described^8^. Labeling of cells with CFSE was as per manufacturer’s instructions (C34554, Life Technologies).

For co-culture of hESCs and smooth muscle cells (SMCs), hESCs were first labeled with CFSE dye and mixed with SMCs at a 5:1 ratio in Smooth Muscle Growth Supplemented (SMGS; S-007-25, Life Technologies) medium. Cells were then seeded on collagen IV (Sigma) coated plates supplemented with Thiazovivin and incubated for 30 mins at 37 ^°^C in 5% CO_2._ After changing the medium to plain SMGS, the cells were taken for imaging. Medium was changed every 24 hours.

For differentiation studies, hESCs were first exposed to mTeSR for 24 hours and then were exposed to DMEM medium (Invitrogen) supplemented with 20% fetal bovine serum (Invitrogen). Medium was changed every 24 hours.

For differentiation to vascular progenitors, methods were as described by Wang *et al.*^14^. Briefly, cells were exposed in a chemically defined medium to 50 ng/ml of Activin A and BMP4 for 2days followed by VEGF and bFGF for 6days.

### FACS sorting of ECAD+/TRA160+ cells

hESCs were pretreated with Rock inhibitor (Y-27632, Cellagen Technology) for 30 mins and then exposed to Accutase ((Innovative Cell Technologies, Inc, San Diego, CA) supplemented with Rock inhibitor for 2–3 mins. Accutase was diluted ten-fold with Calcium – Magnesium free PBS and the suspension was gently flushed several times by 1 ml pipette tip and spun down (300 g, 5 minutes). Cells were resuspended in a small volume of mTeSR supplemented with Thiazovivin and placed in the incubator to recuperate for 30 minutes. After recuperation period, 10 ml FACS buffer (PBS + 0.5% FBS (BSA) + 2 mM EDTA) was added and the suspension was filtered on a 40 μm filter. Cells were spun down again, counted, and resuspended in appropriate volume of buffer for FACS sorting (10^8^ cells maximum in 300 μl FACS buffer). Cells were blocked with mouse IgG (Invitrogen) for 15 minutes and stained for ECAD (Invitrogen) and TRA160 (BD) for 30 minutes on ice. Stained cells were washed with buffer and centrifuged at 300 g for 10 minutes. Pellets were resuspended in 200ul for analysis and 1 ml for sorting. Dual labelled cells were sorted and analyzed on BD FACSAria III cell sorter with FACSDiva Software. Post sorting, the cells were platted on matrigel coated plates in mTeSR supplemented by thiazovivin.

### Immunofluorescence analysis

For immunocytochemistry, cells were fixed in 4% paraformaldehyde/PBS for 20 minutes, washed twice with 0.1% Tween-20/PBS, and blocked with 4% goat or donkey serum in PBS for 30 min—all procedures were done at room temperature. After fixation, the cells were permeabilized with 1% Triton-X/PBS for 30 minutes at room temperature for nuclear or cytoplasmic staining. Subsequently, cells were incubated with primary antibody at room temperature for 1 hour or overnight at 4 °C. The cells were washed with 0.1% Tween-20/PBS three times before secondary antibody was added and incubated for 1 hour at room temperature. Finally, the cells were rinsed with 0.1% Tween-20/PBS three times and counterstained with DAPI (1 μg/ml).

Primary antibodies and dilutions used were as follows: OCT4 (1:200; Santa Cruz), TRA1-60 (1:200; Millipore), TRA1-81 (1:200; Millipore), CD31 (1:100, DAKO) and ECAD (1:200, Conjugated Alexa fluor 647, BD). Secondary antibodies were raised in goat with conjugates: Alexa 488-conjugated anti-rabbit IgG (1:300, Invitrogen), Alexa 594-conjugated anti-rabbit IgG (1:300, Invitrogen), Alexa 488-conjugated anti-mouse IgG (1:300, Invitrogen), Alexa 594-conjugated anti-mouse IgG (1:300, Invitrogen), Alexa 488-conjugated anti-mouse IgM (1:300, Invitrogen) and Alexa 594-conjugated anti-rat IgM (1:300, Invitrogen).

### Cell Imaging

Nikon Biostation IM-Q was used for imaging cells. The Biostation IM-Q is a compact cell incubation and monitoring system that maintains a stable incubation environment (37 °C, 5% CO_2,_ 100% humidity). Forty eight fields of view were acquired every 10 mins for 96 hours. Media changes were done every 24 hrs using the perfusion system on the Biostation. Acquired images were processed using the CMT algorithms.

### Heat Map

The clustergram function in Matlab 2010b—bioinformatics toolbox ver.3.6 was used to generate the dendrogram and heat map. The clustergram function uses Euclidean distance metric and hierarchical clustering with average linkage to generate the hierarchical tree. It clusters first along the columns (producing row-clustered data), and then along the rows in the matrix data. Before clustering the data was standardized along the columns such that the values had a mean of 0 and the standard deviation of 1.

## Additional Information

**How to cite this article**: Phadnis, S. M. *et al.* Dynamic and social behaviors of human pluripotent stem cells. *Sci. Rep.* **5**, 14209; doi: 10.1038/srep14209 (2015).

## Supplementary Material

Supplementary movie 1

Supplementary movie 2

Supplementary movie 3a

Supplementary movie 3b

Supplementary movie 4a

Supplementary movie 4b

Supplementary movie 5

Supplementary movie 6

Supplementary movie 7

Supplementary movie 8

Supplementary movie 9

Supplementary movie 10

Supplementary movie 11

Supplementary movie 12

Supplementary Information
