# Supplementary material for: Dynamic and social behaviors of human pluripotent stem cells
Source: Sci Rep. 2015 Sep 18;5:14209. doi: 10.1038/srep14209 (PMC4585647; doi:10.1038/srep14209)
Supplement: Supplementary Information [file srep14209-s15.pdf]

Dynamic and social behaviors of human pluripotent stem cells

Smruti M. Phadnis[1,2,3,8], Nathan O. Loewke [4,5#], Ivan K. Dimov[1#], Sunil Pai[1#], Christine E. Amwake [4,5], Olav Solgaard[4,5], Thomas M. Baer[6*], Bertha Chen[3*], Renee A. Reijo Pera[1, 2,3,7]

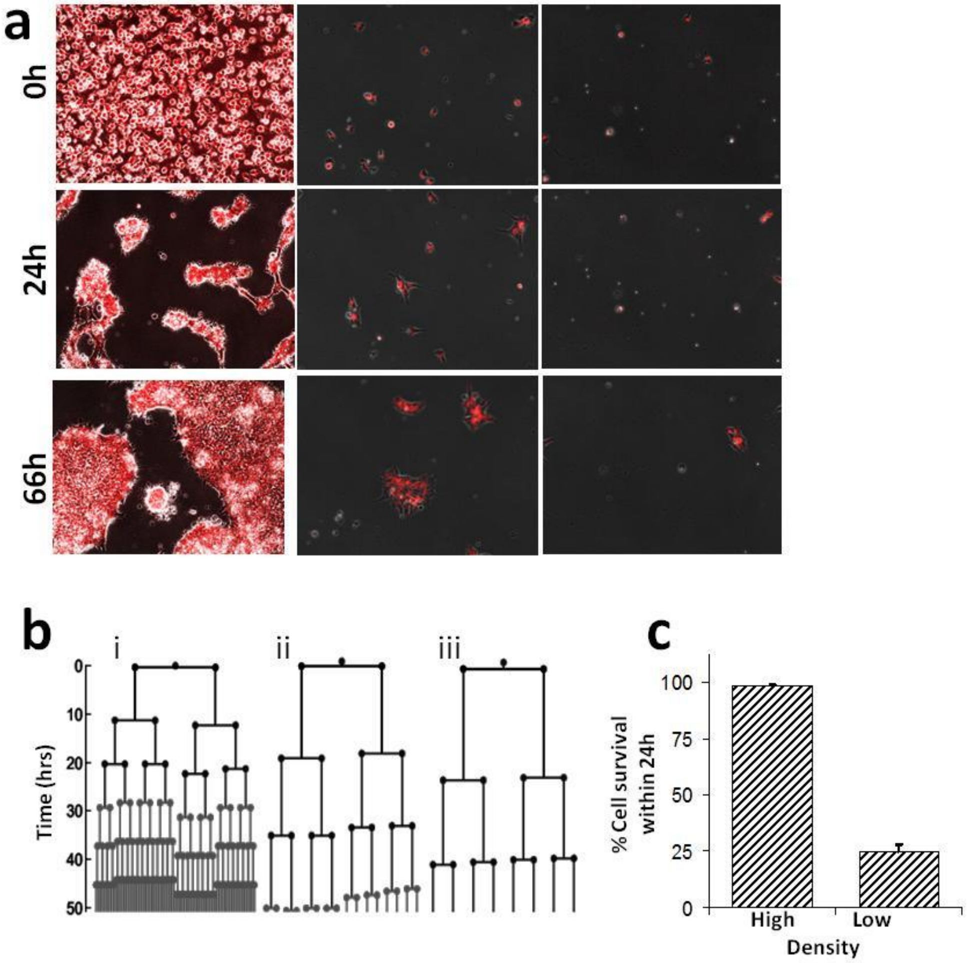

Supplementary Figure S1. Dynamics of hPSCs seeded at different densities

(**a**) Snap shot of human embryonic stem cells (hESCs) seeded at different densities and time (**Supplementary Movie 1, 2 and 3**). (**b**) Representative tree plot derived from manual tracking of mitotic events (and/or number of nuclei) in single hESCs in growth (undifferentiated) conditions and seeded at (i) high density, (ii) medium density, (iii) low density, for 96 hrs. (**c**) Percent cell survival for first 24hrs for cells seeded at high and low densities (n=6 fields of view with around 800 cells each in high density and 25-30 cells at low density).

Input

- Phase contrast images

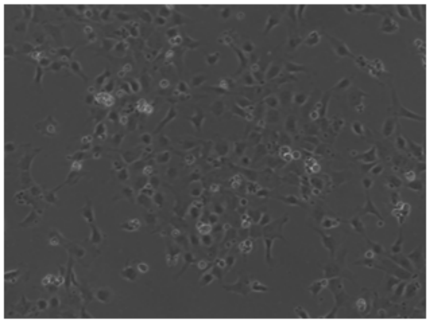

Create Binary Mask

- Research of Kanade group (Carnegie Mellon)
- Based on phase contrast optics
- Fluorescence is simpler

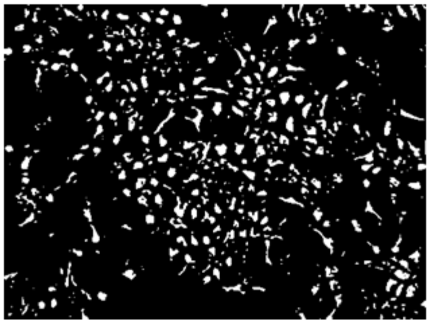

Set Cell Boundaries

- User controls boundaries
- Can eliminate small artifacts seen above

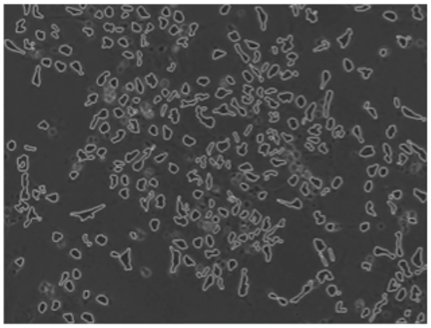

Compute Tracks and Cell Feature Information

- Determine all required cell data from tracks
- Fit physical models to experimental data

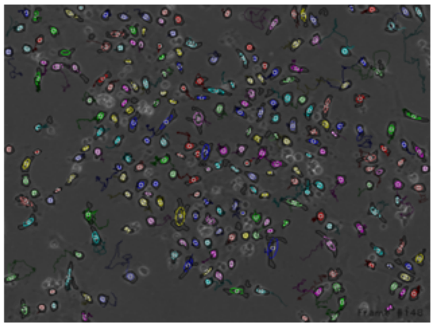

Export to Text File for Post-processing

Variables:

- Cell size/shape
- Cell location/speed
- Cell features
- Neighbor properties
- Cluster formation
- Mitotic events

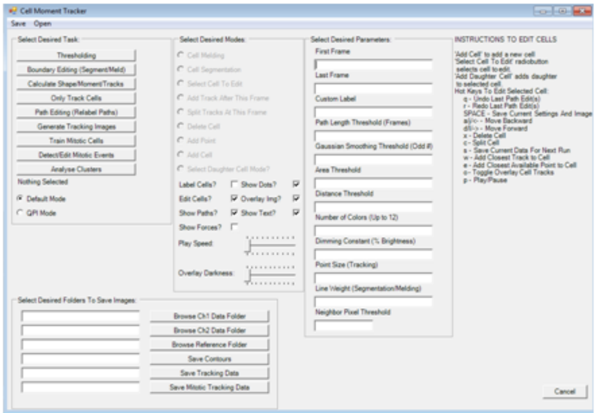

Supplementary Figure S2. Flow Diagram for Cell Moment Tracker (CMT) The overall purpose of CMT is to act as a user interface to analyze large time-lapse data sets of cells. CMT can save prior work or export for post processing, accurately track cells, and compute information about cell shape and migration patterns. Currently, the program has been configured to study cluster formation and morphologies of cells to better understand social behaviors of cells. (**Supplementary Movie 5**)

Binary Mask

Using optimization methods developed by the Takeo Kanade image analysis group at Carnegie Mellon[1], phase contrast images were analyzed to find where cell boundaries are located. From this, a binary mask, an image solely constructed out of black and white pixels, is created to differentiate regions where cells are located from the background of the image. This step is the most crucial step in CMT program because proper cell boundary detection is crucial for the collection of important data about cell morphology and migration patterns

Boundary Error Correction

When dead cells or artifacts are detected, the user can often edit these by adjusting parameters such as the minimum cell size in the image. If the cell boundaries are over-fitted (which may result in overly segmented cell detection), a Gaussian point spread function is applied across the entire image so that the boundaries are smoother and cells are properly detected. CMT also offers options to manually edit the boundaries of cells in case cell or cell-cluster morphology is highly valued in a given experiment.

Computing Tracks and Cell Feature Information

After cell boundaries are detected, a nearest temporal neighbor cell detection method is performed to generate cell migration tracks. Additionally, CMT computes cell shape information such as elliptic Fourier harmonics and spherical harmonics, which can be used to model cell behavior quantitatively. Error correction tools are provided for cell tracking errors or manually detecting mitotic events.

Saving and Post-processing Data

CMT provides a very user-friendly interface to save old data, so that edits can be made on a given data set over a period of several days. Additionally, after computing individual cell features, the program can export the data along with some important outputs about each cell's environment (such as the number of cell neighbors, eccentricity of the cells, and minimum number of neighbors). Using the mitotic detection feature, the program can construct a generation mapping based on mitotic events over the course of a time-lapse video. The ultimate goal will be to correlate the generation number and cell environment to the individual cell's behaviors.

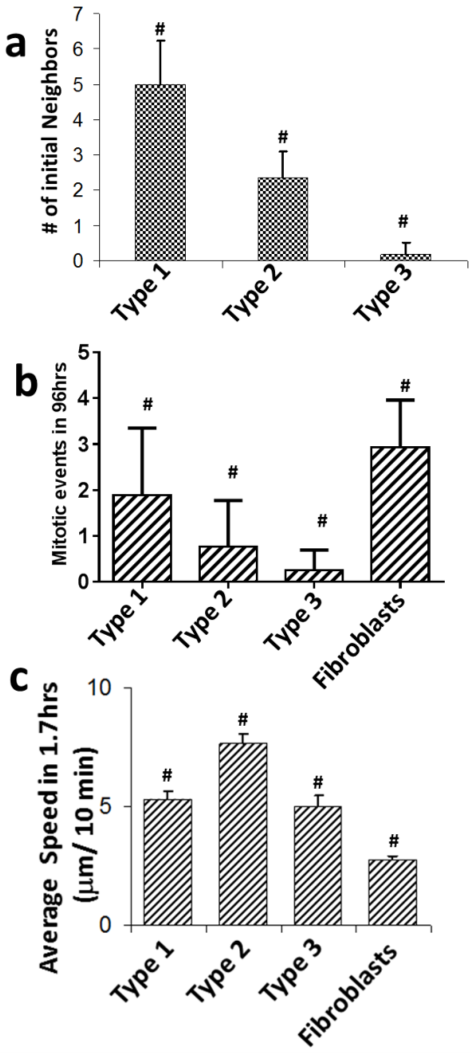

Supplementary Figure S3. Characteristic features extracted from CMT.

(**a**) Number of neighbors in the first frame (time 0, right after attachment to Matrigel) denoted as number of initial neighbors for all the three types of cells (n~20 to 40 cells). (**b**) Number of mitotic events in all the three types of cells (n~40-60 cells) compared with human BJ fibroblasts (n=30) and (**c**) Average speed of cells in first 100mins after attachment to matrigel coated surface (n~100-150 cells each). # indicates two way Anova with $p < 0.0001$

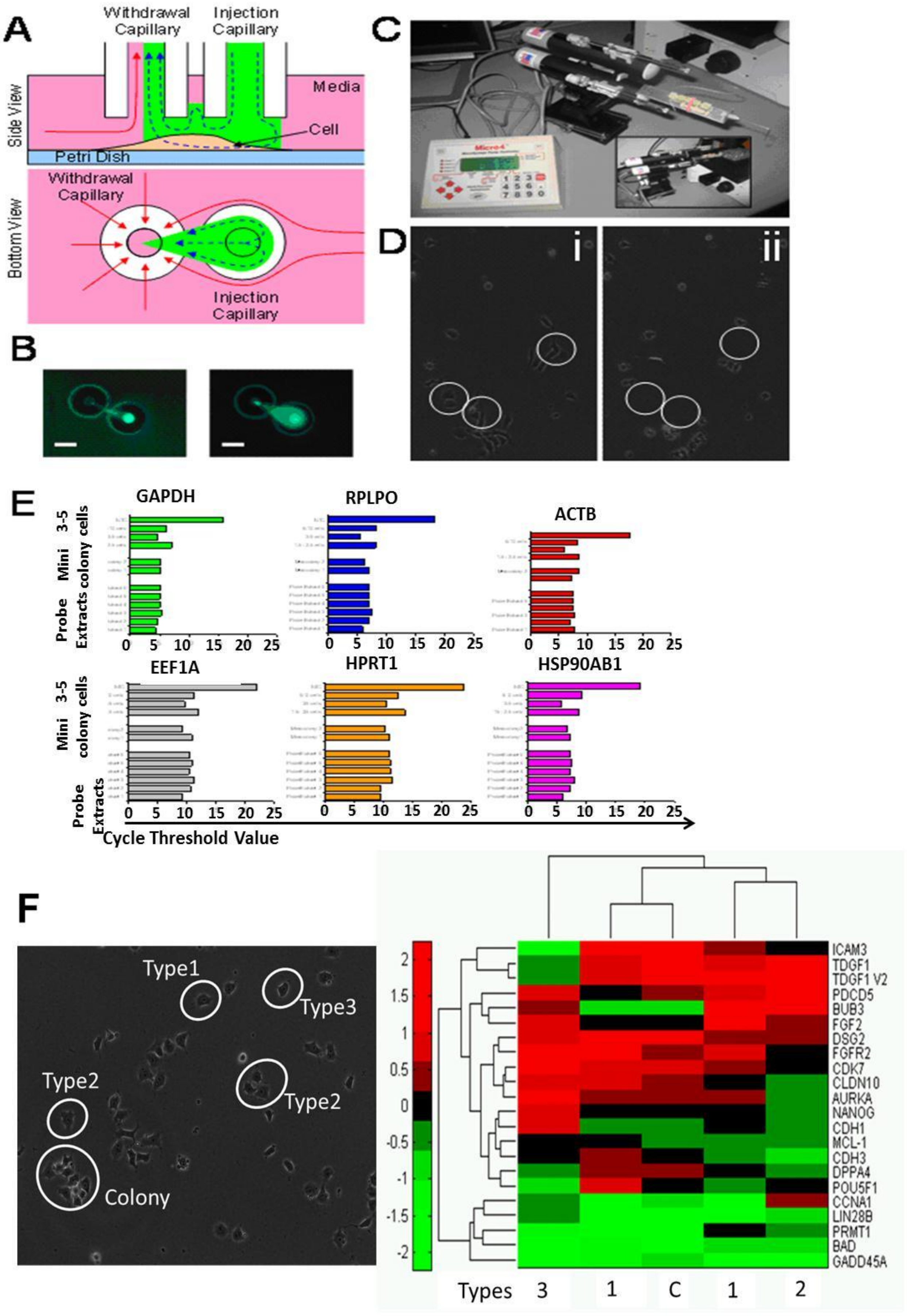
A
Withdrawal Capillary
Injection Capillary
Media
Side View
Cell
Petri Dish
Withdrawal Capillary
Bottom View
Injection Capillary
B
C
D
i
ii
E
GAPDH
RPLPO
ACTB
EEF1A
HPRT1
HSP90AB1
3-5 cells
Mini colony
Probe Extracts
0 5 10 15 20 25
Cycle Threshold Value
F
Type1
Type3
Type2
Type2
Colony
2
1.5
1
0.5
0
-0.5
-1
-1.5
-2
ICAM3
TDGF1
TDGF1 V2
PDCD5
BUB3
FGF2
DSG2
FGFR2
CDK7
CLDN10
AURKA
NANOG
CDH1
MCL-1
CDH3
DPPA4
POU5F1
CCNA1
LIN28B
PRMT1
BAD
GADD45A
Types 3 1 C 1 2

Supplementary Figure S4. Microfluidic probe cell extraction (**a**) Operational principle. The probe uses two capillaries with an inner diameter of 100 um. One capillary injects fluid (enzyme) and the other withdraws it. When the injection to withdrawal ratio is 1:7 or more and the probe is less than 100 um above the surface, hydrodynamic forces form a laminar flow, thus preventing the injected fluid from mixing with the media. Once the fluid injection is stopped it can be completely removed from the dish with minimal contamination. This allows the formation of a zone of influence next to the probe where the injected fluid is present (indicated by the green area). When the probe is placed above a target cell on the culture dish, it gets exposed to the injection fluid whereas the neighboring cells hardly sense the fluid. In this study, cells (imaged for 100 mins and analyzed by CMT) were exposed to accutase in a similar fashion and we were able to selectively detach the cells of interest without disturbing the surrounding cells. When the cells are detached, the inlet flow is stopped and the flow is reversed and the same capillary used to withdraw the cells. The probe is then removed from the dish and the cells are injected into an RT-PCR mixture for gene expression analysis. This entire process is documented under a phase contrast microscope. (**b**) Micrographs of the probe while it is injecting FITC dye. In the left image the injection to withdrawal ratio is 1:30 while in the right image the injection to withdrawal ratio is 1:8. This ratio controls the area of influence of the injected fluid. As the ratio decreases, so does the area of influence. Scale bars are 100 μm. (**c**) Image of the microfluidic probe setup that includes two syringe pumps mounted on a XYZ micromanipulator. The pumps are connected to the 100 μm internal diameter fused silica capillaries using micro tight union connectors. Inset shows how the probe is mounted next to an inverted phase contract microscope for cell extraction. (**d**) i. Phase contract image of H9 cells. The white circle indicates the cells that will be extracted. ii. Phase contract image after the cells were extracted. (**e**) Housekeeping gene expression assays using RT-qPCR were performed to evaluate the effect of the microfluidic probe extraction method on the extracted cell's mRNA abundance. As compared to limited dilution of single cell suspensions, the housekeeping gene abundance is relatively unchanged. This indicates that the microfluidic probe extraction method should not alter the cell's measured mRNA abundance. (**f**) Preliminary RT-qPCR analysis showing average expression of certain genes in extracted cells. Type 1 cells seem to cluster well cells in a colony (C) and also with Type 2 cells. Type 3 cells show a distinct separation. Gene expression normalized to *GAPDH*, *CENTB3*, *EEF1α* and *CNNTB1*. Data represent mean ± SEM (n=2 technical replicates).

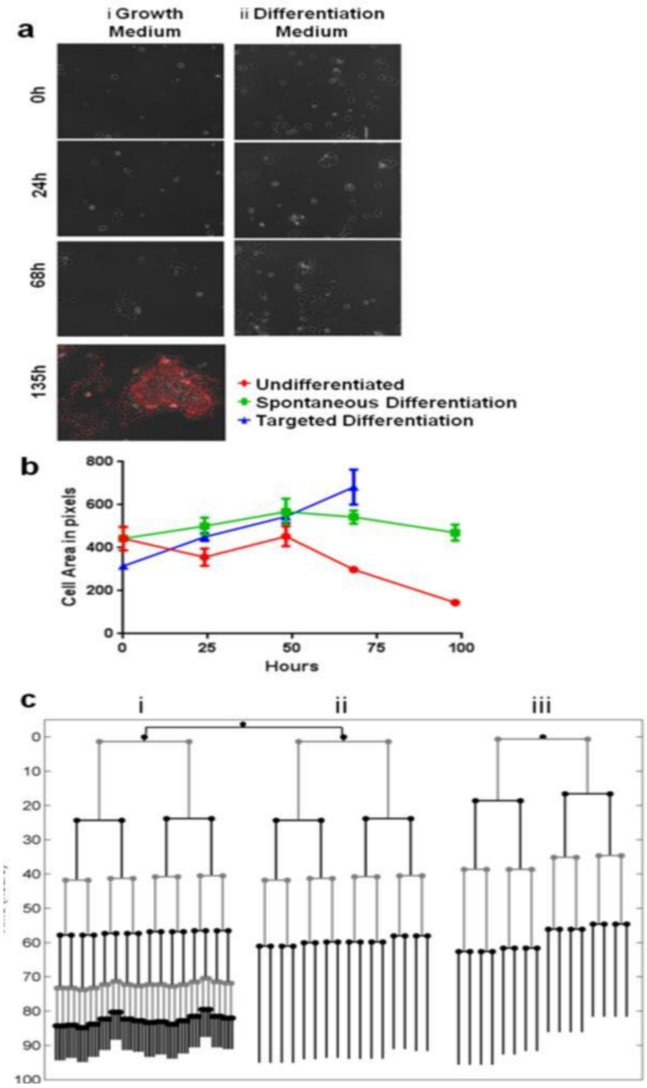

Supplementary Figure S5. Dynamics during early differentiation of hESCs.

(a) Snapshot of cells seeded in (i) growth medium (Supplementary Movie 9) and (ii) differentiation medium (Supplementary Movie 10). (b) Rather than aggregating to form a tight colony, the cells accumulated cytoplasm, thereby increasing cell surface area (n=15). Starting from a single cell, two daughter cells were traced for about 96 hours in medium supporting differentiation or pluripotency. (c) Representative tree lineage tracing of the daughter cells shows spontaneous differentiation in the progeny of (ii) one daughter cell, and maintenance of pluripotency in the progeny of the (i) second daughter cell. (iii) Representative lineage plot of hESCs exposed to differentiation medium indicates lengthier cell cycle as compared to the pluripotent cells (i).

Movie Legend:

Supplementary movie 1: hESCs seeded at high density (150,000 cells/cm^2).

Supplementary movie 2: hESCs seeded at mid density (15,000 cells/cm^2).

Supplementary movie 3a: hESCs seeded at low density (1,500 cells/cm^2).

Supplementary movie 3b: CMT tracking of Movie 3a.

Supplementary movie 4a: hESCs seeded at low density (related to Figure1 in the manuscript).

Supplementary movie 4b: CMT tracking of the movie 4a.

Supplementary movie 5: Human fibroblasts seeded at 1,500 cells/ cm^2.

Supplementary movie 6: $ECAD^{+}TRA160^{+}$ show clonal propagation

Supplementary movie 7: Co-culture of hESCs and smooth muscle cells.

Supplementary movie 8: Spontaneous differentiation of hESCs into fibroblasts in pluripotent medium.

Supplementary movie 9: Targeted differentiation of hESCs into fibroblast in medium supplemented with 20% fetal bovine serum.

Supplementary movie 10: Targeted differentiation of hESCs into vascular progenitors.

Supplementary movie 11: HUF43c5_trisomy12 seeded at 1,500 cells/cm^2.

Supplementary movie 12: Co-culture of normal karyotype hESCs (H9) and abnormal karyotype (HUF43c5_trisomy 12).

<u>Supplementary Methods:</u>

Microfluidic probe for cell extraction

As shown in **Supplementary Fig. S4**, a microfluidic probe was built by mounting two syringe pumps (ULTRA MICRO PUMP 3, WPI Inc., USA) with a 500 μL (for withdrawal) glass syringe (cat. # 7640-01, Hamilton, USA) and a 250 μL (for injection) glass syringe (cat. # 7639-01, Hamilton, USA) onto an XYZ micromanipulator (MD4-M3-L, WPI Inc. USA). The 500 μL syringe was connected to a 100 μm internal diameter fused silica capillary (TSP100170, Polymicro Technologies, UK) while the 250 μL syringe was connected to a 50 μm internal diameter fused silica capillary (TSP050150, Polymicro Technologies, UK) using needles 25 gauge needles (cat. #7732-05, Hamilton, USA) and Micro Tight Union Peek connectors (P-720, Upchurch Scientific, USA). The two fused silica capillaries were routed trough a 90% angle through a gauge 16 needle that was bent. This keeps the two capillaries into conformal contact and makes them come into the petri dish at a right angle to the media surface.

The probe is initially calibrated by first loading a blue food dye into the injection syringe. Then all the capillaries and tubes are primed with media. The probe is mounted on an inverted phase contrast microscope such that the two capillaries are centered with the microscope objective. A cell free medium loaded petri dish is placed on the microscope. The probe is lowered into the dish such that the capillary tips are approximately 30-100 μm above the dish surface and fully submerged into media. The injection and withdrawal flow rates are adjusted until a clear area of influence is seen demarcated by the dye (see **Supplementary Fig. S4b**). The dye is

removed from the syringe and accutase is loaded after the syringe is thoroughly washed with sterile di-H_2O. Then the test dish was replaced with the cultured cell's dish. The cells of interest are identified and positioned in the center of the field of view. The probe is lowered into the dish and positioned right above the cell of interest, such that the cell is right below the injection capillary. The injection and withdrawal flow are initiated and the cell is monitored for dislodgement. As soon as the cell is released from the dish surface the flow is stopped. Then the injection capillary used to withdraw the loose cell by reversing the flow in the corresponding syringe pump. As soon as the cell is removed the flow is stopped. The probe is removed form the pertri dish and placed into 5 μL of 2x RT-qPCR mixture. 5 μL is ejected from the injection capillary into the RT-qPCR mixture this would include the extracted cell. The RT-qPCR reaction can be run the corresponding primers and probes for gene expression analysis.

Supplementary Reference

1 Yin, Z., Kanade, T. & Chen, M. Understanding the phase contrast optics to restore artifact-free microscopy images for segmentation. *Med Image Anal* **16**, 1047-1062, (2012).
